# Mutant *IDH* and non-mutant chondrosarcomas display distinct cellular metabolomes

**DOI:** 10.1186/s40170-021-00247-8

**Published:** 2021-03-24

**Authors:** Sinthu Pathmanapan, Olga Ilkayeva, John T. Martin, Adrian Kwan Ho Loe, Hongyuan Zhang, Guo-Fang Zhang, Christopher B. Newgard, Jay S. Wunder, Benjamin A. Alman

**Affiliations:** 1 Developmental and Stem Cell Biology, Hospital for Sick Children, Toronto, ON Canada; 2 Institute of Medical Science, University of Toronto, Toronto, ON Canada; 3 Department of Pharmacology & Cancer Biology, Duke University, Durham, NC USA; 4 Sarah W. Stedman Nutrition and Metabolism Center and Duke Molecular Physiology Institute, Duke University Medical Center, Durham, NC USA; 5 Department of Orthopaedic Surgery, Duke University, 311 Trent, Durham, NC 27710 USA; 6 Lunenfeld-Tanenbaum Research Institute, and the University Musculoskeletal Oncology Unit, Mount Sinai Hospital, Toronto, ON Canada

**Keywords:** Chondrosarcoma, Metabolism, Mutant IDH, Cancer, Genetic mutation, Amino acids, Acylcarnitines, TCA cycle, Glycolysis

## Abstract

**Background:**

Majority of chondrosarcomas are associated with a number of genetic alterations, including somatic mutations in isocitrate dehydrogenase 1 (*IDH1*) and *IDH2* genes, but the downstream effects of these mutated enzymes on cellular metabolism and tumor energetics are unknown. As *IDH* mutations are likely to be involved in malignant transformation of chondrosarcomas, we aimed to exploit metabolomic changes in *IDH* mutant and non-mutant chondrosarcomas.

**Methods:**

Here, we profiled over 69 metabolites in 17 patient-derived xenografts by targeted mass spectrometry to determine if metabolomic differences exist in mutant *IDH1*, mutant *IDH2*, and non-mutant chondrosarcomas. UMAP (Uniform Manifold Approximation and Projection) analysis was performed on our dataset to examine potential similarities that may exist between each chondrosarcoma based on genotype.

**Results:**

UMAP revealed that mutant *IDH* chondrosarcomas possess a distinct metabolic profile compared with non-mutant chondrosarcomas. More specifically, our targeted metabolomics study revealed large-scale differences in organic acid intermediates of the tricarboxylic acid (TCA) cycle, amino acids, and specific acylcarnitines in chondrosarcomas. Lactate and late TCA cycle intermediates were elevated in mutant *IDH* chondrosarcomas, suggestive of increased glycolytic metabolism and possible anaplerotic influx to the TCA cycle. A broad elevation of amino acids was found in mutant *IDH* chondrosarcomas. A few acylcarnitines of varying carbon chain lengths were also elevated in mutant *IDH* chondrosarcomas, but with minimal clustering in accordance with tumor genotype. Analysis of previously published gene expression profiling revealed increased expression of several metabolism genes in mutant *IDH* chondrosarcomas, which also correlated to patient survival.

**Conclusions:**

Overall, our findings suggest that *IDH* mutations induce global metabolic changes in chondrosarcomas and shed light on deranged metabolic pathways.

**Supplementary Information:**

The online version contains supplementary material available at 10.1186/s40170-021-00247-8.

## Background

Over the past decade, reprogramming of cellular metabolism has gained attention as an emerging hallmark of cancer [1], but metabolic studies of bone and cartilage tumors have been limited. Chondrosarcomas are the most common malignancy of cartilage. They are associated with a number of genetic alterations including somatic mutations in isocitrate dehydrogenase 1 (*IDH1*) and *IDH2* [2, 3]. *IDH* mutations have been identified in 38–70% of chondrosarcoma cases [4] and for this reason, *IDH* has been identified as a potential driver mutation. However, 52–87% of the benign precursor lesion, enchondromas, also harbor *IDH* mutations [4], suggesting that the abnormal *IDH* genes could potentially orchestrate early events in chondrosarcoma formation. These mutations are also found in several neoplasms including gliomas, glioblastomas, and acute myeloid leukemia [3].

*IDH1* and *IDH2* are located in the cytosol and mitochondria respectively, and catalyze the oxidative decarboxylation of isocitrate to α-ketoglutarate (α-KG) [5]. The mutant enzyme loses its ability to convert isocitrate to α-KG, and instead gains a neomorphic function to produce D-2-hydroxyglutarate (D-2HG), which has been referred to as an “oncometabolite” [6]. *IDH* mutations have been reported to be involved in epigenetic remodeling [7], stabilization of hypoxia-inducible factor 1a (HIF1α) [8], and regulation of alternative energetic pathways such as fatty acid metabolism [9].

Here, we have utilized a targeted metabolomics approach [10] to uncover potential metabolic differences between mutant *IDH* and non-mutant chondrosarcomas. Specifically, we compared metabolomic profiles of 4 *IDH* non-mutant chondrosarcomas with 8 mutant *IDH1* and 5 mutant *IDH2* chondrosarcomas (Table 1) using targeted gas chromatography-mass spectrometry (GC/MS) and flow-injection tandem mass spectrometry (MS/MS) methods [10]. By measuring a large number of metabolites, profiles of chondrosarcoma tissues were constructed to comprehensively cover a number of biological processes and metabolic pathways. This approach has been used by others to identify novel metabolic biomarkers and mechanisms in cancer, stem cells, and healthy tissues [10–13]. Our results highlight a number of metabolomic alterations and differentially regulated metabolic pathways in mutant *IDH* compared with non-mutant chondrosarcomas.

**Table 1:** Sequence results and clinicopathological information of chondrosarcomas used for metabolomics analysis

Genotype	Mutation	Tumor grade	Chondrosarcoma subtype	Metastatic status	Recurrence
*IDH1/2*-WT	WT	2	Central	No Metastasis	No
*IDH1/2*-WT	WT	2	Central	No Metastasis	No
*IDH1/2*-WT	WT	2	Central	No Metastasis	No
IDH1/2-WT	WT	1	Central	No Metastasis	No
Mutant *IDH1*	R132C	3	Central	Later Metastasis	Yes
Mutant *IDH1*	R132C	3	Central	Later Metastasis	Yes
Mutant *IDH1*	R132C	2	Central	No Metastasis	No
Mutant *IDH1*	R132C	3	Central	No Metastasis	No
Mutant *IDH1*	R132G	1	Clear Cell	Later Metastasis	Yes
Mutant *IDH1*	R132G	2	Dedifferentiated	Later Metastasis	Yes
Mutant *IDH1*	R132H	3	Clear Cell	Metastasis at Presentation	No
Mutant *IDH1*	R132H	3	Dedifferentiated	Metastasis at Presentation	No
Mutant *IDH2*	R172G	3	Dedifferentiated	Later Metastasis	Yes
Mutant *IDH2*	R172M	3	Dedifferentiated	Metastasis at Presentation	No
Mutant *IDH2*	R172M	3	Dedifferentiated	Metastasis at Presentation	No
Mutant *IDH2*	R172S	3	Dedifferentiated	Later Metastasis	Yes
Mutant *IDH2*	R172S	3	Dedifferentiated	Metastasis at Presentation	No

## Methods

The aim of this study was to assess the metabolic differences between mutant *IDH* and non-mutant chondrosarcomas. To identify and quantify metabolites in patient-derived chondrosarcoma xenograft tissue extracts, we performed targeted GC/MS or flow-injection MS/MS methods.

### Patient-derived xenograft establishment

All human chondrosarcoma samples were handled according to the ethical guidelines of the host institutions. With institutional review board (IRB) approval, human chondrosarcoma tumor samples were obtained fresh from surgery. Within 24 h, tumors were cut into 2-mm^3^ pieces in sterile conditions and were subcutaneously implanted into the flank region of interleukin-2 receptor gamma chain (gamma)-null NOD/SCID (NSG) mice. Tumors were excised once maximal tumor capacity of 2.5 cm was reached. An important consideration in studying tumor metabolomics in patients is variability due to differences in diet, time of surgery, and the presence of medical comorbidities. To control for these patient-specific factors, we established patient-derived chondrosarcoma xenografts in immunodeficient mice. This allowed us to study chondrosarcoma tumor metabolism by eliminating these confounding factors.

### Sequencing

Chondrosarcoma xenograft tumors were genotyped by extracting genomic DNA using the DNeasy Blood and Tissue Kit (Qiagen) according to the manufacturer’s protocol. Sanger sequencing was performed to analyze the samples for *IDH1* and *IDH2* mutations after PCR amplification on exon 4 of *IDH1* and *IDH2* (Table 1). Sequencing results were analyzed on 4Peaks software to confirm mutational status of each tumor.

### Metabolite profiling

To prepare tumors for metabolomics analysis, tumors were pulverized using a prechilled cell crusher in liquid nitrogen. Approximately 50 mg of tumor tissue was added into a prechilled vial and homogenizing solvent (50% aqueous acetonitrile containing 0.3% formic acid) was added to achieve the desired tissue concentration of 50 mg/ml. Mini-BeadBeater Zirconia-Silicate Beads (BioSpec Products), 0.5 mm, were added to the vial to aid with homogenization. Samples were homogenized using Mini-BeadBeater (BioSpec Products) set to a frequency of 30 oscillations/s for 2 min. Metabolic quantification was performed by gas chromatography-mass spectrometry (GC/MS) or flow-injection tandem mass spectrometry (MS/MS) as described previously [14, 15]. Amino acids, acylcarnitines, and organic acids were analyzed using the stable isotope dilution technique. Amino acids and acylcarnitine measurements were made by flow-injection tandem mass spectrometry using sample preparation methods described previously [16, 17]. The data were acquired using a Waters TQD mass spectrometer equipped with an Acquity^TM^ UPLC system and controlled by MassLynx 4.1 operating system (Waters, Milford, MA). Organic acids were quantified using methods described previously [18] employing Trace Ultra GC coupled to ISQ MS operating under Xcalibur 2.2 (Thermo Fisher Scientific, Austin, TX). Data analysis of metabolites was performed using Student’s *t*-test (*p* < 0.05).

### UMAP analysis

To evaluate relative similarities between each chondrosarcoma, data was processed using Uniform Manifold Approximation and Projection [19] (UMAP) for dimensional reduction and visualization. In total, 7 organic acids, 17 amino acids, and 45 acylcarnitines were screened and separate UMAP projections were performed for each metabolite group (i.e., three groups with 17 tumors and 7, 17, or 45 dimensions). First, data was pre-processed by scaling the mean and variance (*μ* = 0 and *σ* = 1). Then, UMAP was performed for 2D visualization (R version 4.0.0 [20]); Package “Seurat” (Stuart, Butler, Hoffman, et al., 2019)-function RunUMAP; nearest neighbors: 15, minimum distance: 0.7, distance metric: Euclidean). After UMAP projection, tumors were labeled based on their genotype and evaluated qualitatively.

#### Carbon isotope labeling

Chondrosarcoma cells were cultured in DMEM with 1000mg/L ^13^C_6_-Glucose in 6-cm cell culture plates for 6 h. A total of 500 μL methanol was used to extract metabolites from each plate. After centrifuging at 12000 rpm for 15 min, supernatant was dried at 37 °C. The dried residues were resuspended in 25 μL of methoxylamine hydrochloride (2% (w/v) in pyridine) and incubated at 40 °C for 1.5 h in a heating block. After brief centrifugation, 35 μL of MTBSTFA + 1% TBDMS was added, and the samples were incubated at 60 °C for 30 min. The derivatized samples were centrifuged for 5 min at 20,000×*g*, and the supernatants were transferred to GC vials for GC-MS analysis. A modified GC-MS method was employed [21]. The injection volume was 1 μL, and samples were injected in splitless mode. GC oven temperature was held at 80 °C for 2 min, increased to 280 °C at 7°C/min, and held at 280 °C for a total run time of 40 min. GC-MS analysis was performed on an Agilent 7890B GC system equipped with a HP-5MS capillary column (30 m, 0.25 mm i.d., 0.25-μm phase thickness; Agilent J&W Scientific, Santa Clara, CA), connected to an Agilent 5977A Mass Spectrometer operating under ionization by electron impact (EI) at 70 eV. Helium flow was maintained at 1 mL/min. The source temperature was maintained at 230 °C, the MS quad temperature at 150 °C, the interface temperature at 280 °C, and the inlet temperature at 250 °C. Mass spectra were recorded in mass scan mode with *m*/*z* from 50 to 700.

### ^13^C-based stable isotope analysis

M0, M1, …, M*n* refers to the isotopologues containing *n* heavy atoms in a molecule. The stable isotope distribution of individual metabolites was measured by GC-MS as described above. The isotopologue enrichment or labeling in this work refers to the corrected isotope distribution [22, 23]. To determine the flux from citrate/isocitrate to α-ketoglutarate, we normalized the % of ^13^C-α-ketoglutarate to the percentage of ^13^C-citrate/isocitrate.

### Gene expression profiling data analysis

Chondrosarcoma gene expression data was previously published by another group in their identification of genes that determine disease progression [24]. Their mRNA microarray data and chondrosarcoma sample information [25] E-MTAB-7264 (https://www.ebi.ac.uk/arrayexpress/experiments/E-MTAB-7265/) were downloaded from the European Bioinformatics Institute database [26] [PMID: 31604924]. Microarray data was normalized using the RMA algorithm with the “oligo” package [PMID: 20688976]. Gene expression data was further scaled for the generation of heatmaps. For survival analysis, samples were grouped into high/low expression based on median gene expression and plotted using the “survminer” package.

## Results

### Mutant IDH1 and IDH2 chondrosarcomas display an elevation of glycolytic and TCA cycle intermediates compared with non-mutant chondrosarcomas

**Fig. 1:**
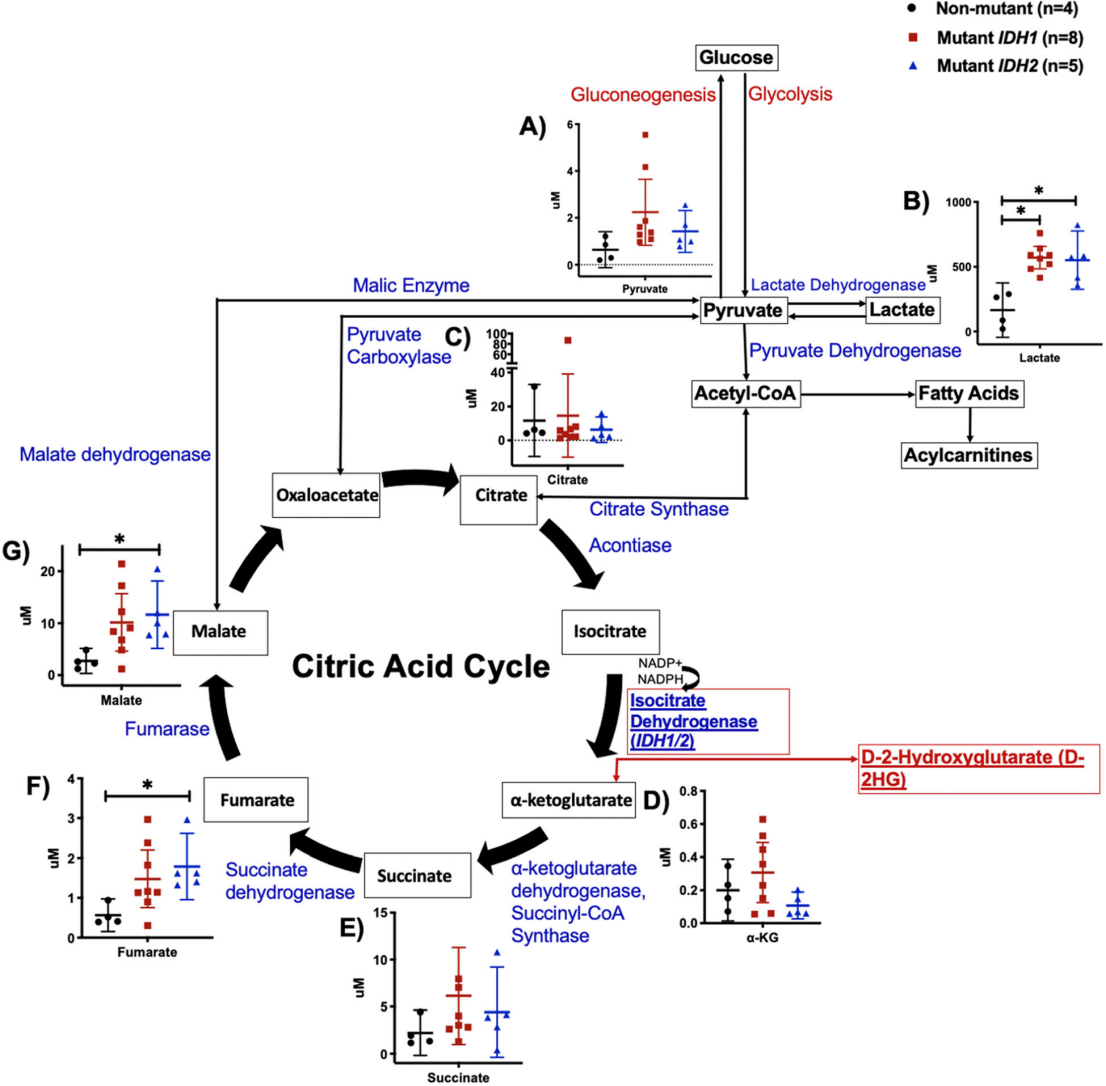
Organic acid quantification of non-mutant, mutant *IDH1*, and mutant *IDH2* chondrosarcoma tissue by gas chromatography/mass spectrometry (GC/MS). Quantification of glycolytic **a** pyruvate, **b** lactate and TCA cycle metabolites **c** citrate, **d** ⍺-ketoglutarate, **e** succinate, **f** fumarate, **g** malate, of non-mutant, mutant *IDH1*, and mutant *IDH2* chondrosarcoma tissue. *p*-value = Student’s *t*-test *p* < 0.05, *: significant *p*-value

To examine whether mutant *IDH* chondrosarcomas display metabolomic differences in comparison with non-mutant chondrosarcomas, we used gas chromatography/mass spectrometry (GC/MS) to profile 7 organic acids generated by glycolysis and the TCA cycle. Mutant *IDH1* and *IDH2* chondrosarcomas displayed a 3.5-fold elevation of lactate (Fig. 1b), coupled with a trend for increase of pyruvate (Fig. 1a), suggestive of enhanced glycolytic metabolism of glucose in mutant *IDH* chondrosarcomas.

Five TCA cycle intermediates were also quantified to determine if differences may exist in mitochondrial carbon cycling. Three intermediates generated in the latter part of the TCA cycle, succinate, fumarate, and malate were increased in mutant *IDH1* and *IDH2* chondrosarcomas compared with non-mutant chondrosarcomas (Fig. 1e–g). A different and more complex profile was found for the early TCA cycle intermediates—citrate and α-KG trended to increase in mutant *IDH1* but decrease in mutant *IDH2* chondrosarcomas (Fig. 1c, d). These differences may reflect the role of *IDH1* in the cytosol as a source of NADPH for de novo lipogenesis [27] and other biosynthetic pathways, whereas *IDH2* participates both in oxidative flux of α-ketoglutarate to the later TCA cycle intermediates, as well as reductive carboxylation of α-ketoglutarate to isocitrate and citrate in the mitochondria [28, 29].

Activation of reductive TCA cycle metabolism of glutamine and glutamate via conversion to α-ketoglutarate by the glutamate dehydrogenase reaction and flux to citrate via *IDH2* has been invoked as a pathway for synthesis of biomass in growing cancer cells [29–31]. If *IDH2* mutation impairs reductive flux of glutamine to citrate in chondrocytes, this may help to explain why citrate and α-ketoglutarate are depleted in mutant *IDH2* chondrosarcomas (Fig. 1c, d; Supplemental Figure 2). Perhaps related, it has been suggested that citrate and α-ketoglutarate levels are influenced by multiple events limiting entry of carbon into the TCA cycle [30, 32, 33] including reduced mitochondrial NAD^+^/NADH ratio [30], reduced glutamine metabolism [30, 33], and inactivation of pyruvate dehydrogenase in hypoxic tumors [32].

To examine how mutant *IDH* alters flux through the TCA cycle, we traced [^13^C_6_]-labeled glucose. ^13^C α-KG labeling was reduced at 6 h in mutant *IDH1* and *IDH2* chondrosarcoma cells based on measurements of citrate (M2) and pyruvate (M3) labeling (Supplemental Figure 2). This result clarifies that carbon flux through mutant *IDH* is impaired, explaining depleted α-KG levels (Fig. 1d) in mutant *IDH2* chondrosarcomas.

We next performed UMAP clustering analysis [19] on our metabolite dataset to examine if relative similarities exist between each chondrosarcoma genotype group (Supplemental Figure 1). UMAP projections performed on 7 organic acids revealed distinct clustering patterns in non-mutant and mutant *IDH* chondrosarcomas (Supplemental Figure 1A). Although organic acids in mutant *IDH1* and mutant *IDH2* chondrosarcomas did not cluster separately from one another, they clustered separately from the non-mutant group (Supplemental Figure 1A). UMAP projections reveal that chondrosarcomas of identical genotypes exhibit similar organic acid profiles. Taken together, our data is consistent with the notion that mutant *IDH* chondrosarcomas not only display high glycolytic and TCA cycle intermediates for energy production and maintenance but also display a distinct organic acid profile in comparison with non-mutant chondrosarcomas.

### Global elevation of amino acids in mutant IDH chondrosarcomas in comparison with non-mutant chondrosarcomas

**Fig. 2:**
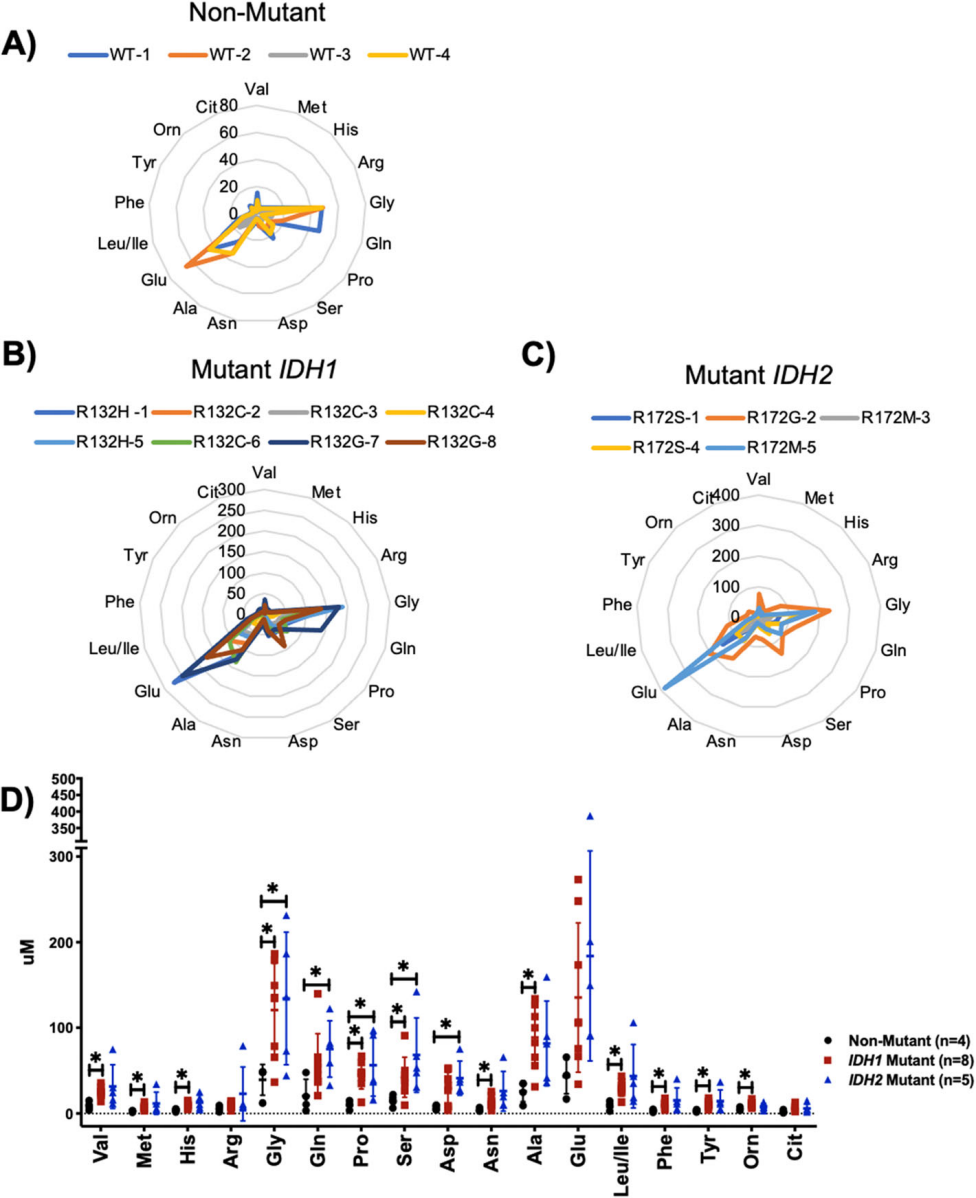
Amino acid profiles of non-mutant, mutant *IDH*, and mutant *IDH2* chondrosarcoma tissue quantified by mass spectrometry (MS/MS) display a global elevation in mutant *IDH* chondrosarcomas. Radar profiles of **a** non-mutant chondrosarcomas, **b** mutant *IDH1* chondrosarcomas, **c** mutant *IDH2* chondrosarcomas, **d** scatter plot of amino acids in each genotype of chondrosarcoma. *p*-value = Student’s *t*-test *p* < 0.05, *: significant *p*-value

We next measured levels of 17 amino acids by MS/MS and constructed amino acid profiles of each patient tumor. We aimed to characterize overlapping and/or distinct patterns of expression in mutant *IDH* and non-mutant chondrosarcomas. Irrespective of mutational status, glycine (Gly), glutamate (Glu), glutamine (Gln), and alanine (Ala) were the most abundant amino acids in chondrosarcomas (Fig. 2a–c). This observation is supported by the sharp peak concentrations as indicated from radar profiles of chondrosarcoma tissues (Fig. 2a–c).

A broad elevation of amino acids was observed in mutant *IDH* chondrosarcomas compared with non-mutant tumors (Table 2, Fig. 2d). Twelve of the 17 amino acids measured (Val, Met, His, Gly, Pro, Ser, Asn, Ala, Leu/Ile, Phe, Tyr, and Orn) were significantly elevated in mutant *IDH1* chondrosarcomas. These same 12 amino acids exhibited clear trends to increase in mutant *IDH2* chondrosarcomas, although only 5 of these (Gly, Gln, Pro, Ser, and Asp) achieved statistical significance (Table 2, Fig. 2d). Mechanisms for this broad increase in amino acids are not revealed by our data, but two possibilities are that mutant *IDH* may activate autophagy [34–36] or protein degradation [37, 38]. We used UMAP clustering analysis to determine if mutant *IDH* chondrosarcomas displayed distinct amino acid profiles compared with non-mutant chondrosarcomas (Supplemental Figure 1B). Although amino acid profiles of mutant *IDH* chondrosarcomas clustered together, two mutant *IDH1* tumors clustered closely to the non-mutant cluster group (Supplemental Figure 1B).

**Table 2:** Fold change analysis reveals global elevation of amino acids in mutant *IDH* chondrosarcomas in comparison with non-mutant chondrosarcomas

Amino acid	Fold change—mutant *IDH1* vs non-mutant Chondrosarcoma	*p*-value	Fold change—mutant *IDH2* vs non-mutant Chondrosarcoma	*p*-value
Glucogenic amino acids				
Val	2.8	0.0015*	3.6	0.1275
Met	2.7	0.0156*	4.5	0.1989
His	2.6	0.0071*	3.4	0.0703
Arg	1.5	0.1729	3.8	0.3309
Gly	3.1	0.0174*	3.4	0.0497*
Gln	2.8	0.0950	3.7	0.0222*
Pro	4.5	0.0021*	5.7	0.0404*
Ser	2.8	0.0502*	4.5	0.0487*
Asp	4.0	0.0639	5.8	0.0125*
Asn	3.0	0.0340*	6.0	0.1083
Ala	3.4	0.0080*	3.2	0.0659
Glu	3.2	0.0680	4.3	0.0594
Ketogenic amino acids				
Leu/ Ile	3.2	0.0021*	4.8	0.1097
Glucogenic & ketogenic amino acids				
Phe	3.3	0.0099*	4.9	0.1459
Tyr	3.3	0.0068*	4.5	0.1403
Other amino acids				
Orn	2.0	0.0128*	1.5	0.2156
Cit	2.8	0.0667	2.8	0.1950

*p*-value = *t*-test *p* < 0.05, italic, bold: significant in *t*-test

### Amino acids involved in neoplastic energy-yielding pathways are elevated in mutant IDH chondrosarcomas

An abundant supply of amino acids is essential for tumors to sustain their proliferation through energy generation, nucleoside synthesis, protein synthesis, and cellular redox homeostasis [38]. For this reason, specific amino acids are coupled to neoplastic metabolic pathways [39]. Anaplerotic metabolism of amino acids regenerates key TCA cycle and glycolytic intermediates that enable carbon flow and ATP production in neoplastic cells. Further examination of our data suggests that several amino acids elevated by *IDH* mutations are critical anaplerotic substrates.

Glutamine (Gln) and glutamate (Glu) are involved in glutamine metabolism to fuel reductive and oxidative TCA carbon cycling. Gln and Glu are converted to α-ketoglutarate by the glutamate dehydrogenase reaction or by glutamate transamination. Gln levels trended to increase by 2.8-fold in mutant *IDH1* chondrosarcomas and achieved statistical significance in mutant *IDH2* chondrosarcomas (Table 2). Glu trended to increase by 3.2-fold and 4.3-fold in mutant *IDH1* and mutant *IDH2* tumors respectively (Table 2). Additionally, proline (Pro), another critical amino acid metabolized via conversion to glutamate and was found to be elevated in mutant *IDH* chondrosarcomas (Table 2). This suggests that mutant *IDH* chondrosarcomas possess a greater reliance of glutamine metabolism.

Alanine (Ala), glycine (Gly), and serine (Ser), which are broadly elevated in mutant *IDH* chondrosarcomas (Fig. 2d, Table 2), enter pathways of energy production via pyruvate, which in turn can be oxidized via pyruvate dehydrogenase or used as an anaplerotic substrate to generate oxaloacetate via pyruvate carboxylase. Aspartate (Asp) and asparagine (Asn), which are also elevated in mutant *IDH* tumors (Fig. 2d, Table 2), act as anaplerotic substrates via their conversion to oxaloacetate by aspartate transaminase. A previous study identified that elevated Asp levels serve as an endogenous metabolite, supporting tumor growth via conversion of Asn to Asp by asparaginase activity [40] and maintenance of Asp pools by electron acceptor activity was found to support cell proliferation [41]. Interestingly, our profile suggests that Asn and Asp are crucial substrates for TCA cycle fueling in mutant *IDH* chondrosarcomas.

Finally, the branched-chain amino acids (BCAAs), valine (Val), leucine (Leu), and isoleucine (Ile), have been implicated in metabolic reprogramming of neoplastic cells to promote cancer growth [42]. Individual Val and Leu/Ile levels were significantly elevated in mutant *IDH1* chondrosarcomas and trended to increase in mutant *IDH2* chondrosarcomas (Table 2). The increase in Val and Leu/Ile in mutant *IDH* chondrosarcomas is associated with similar increases in their transamination by-products Glu and Gln (Fig. 2a–d, Table 2).

### Acylcarnitine species are elevated in mutant IDH chondrosarcomas

Acylcarnitines are generated by metabolism of fatty acids, glucose, and amino acids, and serve as cognate metabolites to acyl-CoAs generated in the mitochondria and cytosol [14]. We measured 45 independent acylcarnitine species ranging from 2 to 22 carbons in length, and with varying degrees of saturation by MS/MS. Even chain species ranging in length from C6 to C22 arise from fatty acid oxidation [14]. Odd chain species such as C3 (propionylcarnitine) and C5 (isovalerylcarnitine) are produced by amino acid catabolism, whereas C4 (butyrylcarnitine) can be derived from fatty acids or amino acids [14]. Acetylcarnitine (C2) is derived from acetyl-CoA, a common degradation product for many metabolic substrates. As in other tissues, acetylcarnitine (C2) is the most abundant acylcarnitine species in chondrosarcomas.

**Fig. 3:**
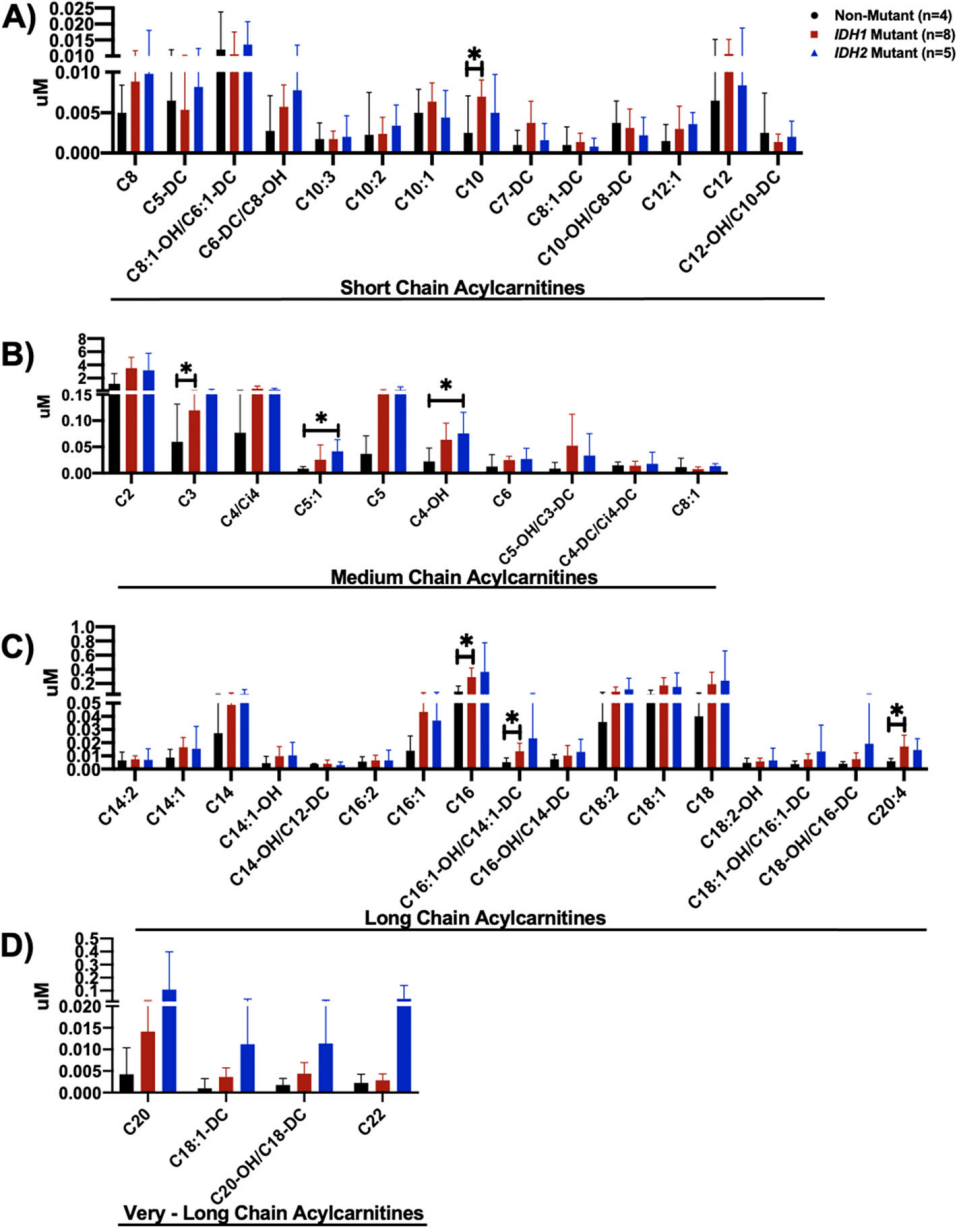
Acylcarnitine profiles of non-mutant, mutant *IDH1*, and mutant *IDH2* chondrosarcoma tissues, quantified by mass spectrometry (MS/MS) display an elevation of species in mutant *IDH* chondrosarcomas. Acylcarnitines classified according to increasing carbon chain length: **a** short-chain acylcarnitines, **b** medium-chain acylcarnitines, **c** long-chain acylcarnitines, **d** very-long-chain acylcarnitines. *p*-value = Student’s *t*-test p < 0.05, *: significant *p*-value

Interestingly, a number of long-chain lipid-derived acylcarnitines were found to be elevated in mutant *IDH* chondrosarcomas compared with non-mutant tumors, including oleate (C18:1), palmitate (C16:0), linoleate (C18:2), and stearate (C18:0) (Fig. 3c, Table 3). Consistent with a profile of increased fatty acid oxidation was the increase in C4-OH acylcarnitine (beta-hydroxy butyryl AC) in mutant *IDH* chondrosarcomas (Fig. 3a, Table 3). C3, C4, and C5 acylcarnitines derived from amino acid catabolism also trended higher in mutant *IDH* chondrosarcomas (Fig. 3a, Table 3).

**Table 3:** Fold change analysis reveals elevated levels of acylcarnitine species in mutant *IDH* chondrosarcomas

Lipid profiling acylcarnitines	Acylcarnitine length	Fold change—mutant *IDH1* vs non-mutant Chondrosarcoma	*p*-value	Fold change—mutant *IDH2* vs non-mutant Chondrosarcoma	*p*-value
Acetyl-CoA derivative					
C2	SC-AC	3.0	0.0510	2.8	0.1189
Amino acid derivatives—odd chain species					
C3	SC-AC	2.0	0.0430*	2.6	0.2937
C5:1	SC-AC	3.0	0.3391	4.9	0.0087*
C5	SC-AC	4.3	0.1445	6.3	0.3449
C5-OH/C3-DC	SC-AC	6.2	0.2576	4.0	0.1920
C5-DC	MC-AC	0.9	0.7707	1.3	0.4642
C7-DC	MC-AC	3.7	0.1326	1.7	0.5192
Fatty acid derivatives—even chain species					
C6	SC-AC	2.0	0.0842	2.2	0.2127
C8:1	SC-AC	0.7	0.3901	1.2	0.7380
C8	MC-AC	1.7	0.0722	1.9	0.2351
C8:1-OH/C6:1-DC	MC-AC	0.9	0.7353	1.1	0.7281
C6-DC/C8-OH	MC-AC	2.0	0.1644	2.8	0.0969
C10:3	MC-AC	1.0	0.9255	1.1	0.8489
C10:2	MC-AC	1.1	0.8987	1.6	0.4553
C10:1	MC-AC	1.3	0.4115	0.9	0.7575
C10	MC-AC	2.9	0.0162*	2.0	0.2869
C8:1-DC	MC-AC	1.6	0.5332	0.7	0.7576
C10-OH/C8-DC	MC-AC	0.8	0.6839	0.6	0.2910
C12:1	MC-AC	1.9	0.4518	2.2	0.0732
C12	MC-AC	1.6	0.2373	1.3	0.7066
C12-OH/C10-DC	MC-AC	0.6	0.3945	0.9	0.8496
C14:2	LC-AC	1.1	0.7881	1.0	0.9626
C14:1	LC-AC	1.9	0.0985	1.7	0.5002
C14	LC-AC	1.8	0.1588	2.2	0.3238
C14:1-OH	LC-AC	2.1	0.2333	2.3	0.2990
C14-OH/C12-DC	LC-AC	1.1	0.7262	0.8	0.6335
C16:2	LC-AC	1.2	0.6409	1.2	0.7771
C16:1	LC-AC	3.1	0.0675	2.6	0.3013
C16	LC-AC	3.3	0.0164*	4.1	0.2330
C16:1-OH/C14:1-DC	LC-AC	2.6	0.0313*	4.5	0.3420
C16-OH/C14-DC	LC-AC	1.4	0.5234	1.8	0.3100
C18:2	LC-AC	2.4	0.2108	3.3	0.3426
C18:1	LC-AC	3.3	0.0734	2.9	0.3638
C18	LC-AC	4.8	0.1059	6.0	0.3753
C18:2-OH	LC-AC	1.3	0.4134	1.6	0.6317
C18:1-OH/C16:1-DC	LC-AC	2.0	0.1328	3.6	0.3811
C18-OH/C16-DC	LC-AC	1.9	0.1788	4.6	0.4159
C20:4	LC-AC	2.9	0.0293*	2.4	0.0883
C20	VLC-AC	3.2	0.1964	24.6	0.4088
C18:1-DC	VLC-AC	3.3	0.0861	9.9	0.3591
C20-OH/C18-DC	VLC-AC	2.8	0.1026	7.0	0.2104
C22	VLC-AC	1.3	0.5074	15.7	0.4142
Amino acid & fatty acid derivatives					
C4/Ci4	SC-AC	5.9	0.1180	3.7	0.0602
C4-OH	SC-AC	2.9	0.0676	3.4	0.0193*
C4-DC/Ci4-DC	SC-AC	1.0	0.9681	1.2	0.7348

*p*-value = *t*-test *p* < 0.05, italic, bold: significant in *t*-test

*SC-AC* short-chain acylcarnitine

*MC-AC* medium-chain acylcarnitine

*LC-AC* long-chain acylcarnitine

*VLC-AC* very-long-chain acylcarnitine

To examine relative differences in acylcarnitine metabolism between mutant *IDH* and non-mutant chondrosarcomas, we compared means of acylcarnitine species in mutant *IDH* tumors to non-mutant tumors and calculated concentration fold changes (Table 3). From this analysis, we found medium- and long-chain lipid-derived acylcarnitines, C10, C16, and C16:1-OH/C14:1-DC significantly elevated in mutant *IDH1* chondrosarcomas compared with non-mutant chondrosarcomas (Table 3). The amino acid-derived acylcarnitines, C5:1 and C4-OH were significantly elevated in mutant *IDH2* chondrosarcomas compared with non-mutant tumors (Table 3).

UMAP clustering analysis of 45 acylcarnitines revealed weak clustering of mutant *IDH* and non-mutant chondrosarcomas (Supplemental Figure 1C). Although majority of mutant *IDH* chondrosarcomas clustered away from the non-mutant group, these clusters were spatially weak clusters (Supplemental Figure 1C). Although there may be some similarities in acylcarnitine profile in mutant *IDH* chondrosarcomas, these similarities are not as pronounced as clustering similarities noted in organic acid and amino acid profiles (Supplemental Figure 1A-C). Clustering analysis of the complete dataset of 69 metabolites revealed distinct clustering of mutant *IDH* chondrosarcomas from non-mutant tumors (Supplemental Figure 1D), emphasizing the notion that mutant *IDH* chondrosarcomas display a distinct metabolome in comparison with non-mutant chondrosarcomas.

### Expression of genes regulating metabolism correlates with prognosis in chondrosarcomas

To determine the significance of our findings in a second chondrosarcoma patient cohort, we explored the expression levels of regulatory genes of metabolic pathways from previously published gene expression and patient survival data [24, 25]. Using normalized microarray gene expression data [24, 25], we found a number of genes involved in various metabolic processes to be elevated in mutant *IDH* tumors (Supplemental Figure 3A). For instance, genes involved in the regulation of glutamine metabolism, *GLS* and *GLUD1*; a biomarker of glycolysis, *LDHA*; and genes involved in TCA cycle regulation, *PDK1*, *PDHB*, *MDH1*, and *SHMT2*; were all significantly elevated in mutant *IDH* chondrosarcomas compared with non-mutant tumors (Supplemental Figure 3A).

The expression levels of a number of these genes significantly correlated with patient survival when analyzed using Kaplan-Meier survival analysis (Supplemental Figure 3B-H). Interestingly, chondrosarcomas with high expression levels of two regulators of acylcarnitine metabolism, *CPT1A* and *FASN*, displayed worse patient outcome (Supplemental Figure 3B-C). High expression of regulatory genes of the TCA cycle (*SDHAF2*, *MDH1*, *MDH2*, and *PDHB*) and glutamine metabolism (*GLUD1*), were also predictive of patient outcome (Supplemental Figure 3E-H, D). Taken together, these data are constant with the notion that specific metabolites and their associated regulatory genes are critical in chondrosarcoma metabolomics, drive tumor aggressiveness, and are predictive of patient survival.

## Discussion

Here we report metabolic differences between mutant *IDH* chondrosarcomas and non-mutant chondrosarcomas across a spectrum of organic acids, amino acids, and acylcarnitines measured by targeted GC/MS and MS/MS mass spectrometry. Mutant *IDH* chondrosarcomas display metabolomes enriched for lactate, late-stage TCA cycle intermediates, multiple amino acids, and a subset of acylcarnitine species. Interestingly, UMAP projection analysis of our metabolite dataset revealed that organic acids and amino acids displayed spatially strong and distinct clustering patterns in accordance with each chondrosarcoma genotype. Upon analysis of all 69 metabolites by UMAP, this revealed spatially distinct clustering in mutant *IDH* chondrosarcomas and non-mutant chondrosarcomas. Thus, from our complete dataset, we were also able to conclude that mutant *IDH* chondrosarcomas display a distinct metabolic profile compared with non-mutant chondrosarcomas.

Metabolic changes can be cell type dependent, and although other studies show metabolic changes due to *IDH* mutations in other cell types, data from these studies do not extend to all cell types. Metabolic changes in cell culture may not reflect changes seen in vivo. This is a particular issue in certain tumor types like chondrosarcomas, where the cell phenotype changes in vitro. Indeed, differences in diet, time of surgery, and the presence of medical comorbidities will cause metabolic changes in tumors. To control for these factors while still analyzing tumors in vivo, we studied xenografts, allowing us to control for various external factors. Thus, our approach allows an analysis of chondrosarcoma tumor metabolism eliminating confounding factors of cell culture and patient variability.

We found an elevation of lactate in mutant *IDH* chondrosarcomas. Conversion of glucose carbon to lactate by the lactate dehydrogenase (*LDHA*) enzyme is a classic signature of cancer metabolism [43]. More importantly, lactate is not only a metabolic by-product of active glycolysis but has many other consequential effects on cancer bioenergetics and carcinogenesis [43]. Glycolytic tumors that produce lactate create an acidic tumor microenvironment which supports metastasis, angiogenesis, and immunosuppression [44]. It has also been demonstrated that neoplastic cells possess the ability to import lactate into the mitochondria to fuel energy production [45]. While a prior study showed elevated *LDHA* activity in chondrosarcomas, our data shows that genotype alters the level of lactate, and as such genotype should be taken into account when considering strategies to target this pathway [46].

Hypoxic tumors that have the ability to thrive in low oxygen environments are associated with lactate accumulation [43, 47]. Interestingly, it has been reported that glucose metabolism and hypoxia-inducible factor (*HIF1a*) levels are elevated in chondrosarcomas and correlated with a higher pathological grade and lower patient survival rate [48]. This suggests that mutant *IDH* chondrosarcomas possess a greater dependence on glycolysis for energy and are metabolically more active compared with non-mutant chondrosarcomas.

Another metabolic alteration caused by a mutated *IDH* enzyme was the broad elevation of amino acids. Specifically, amino acid profiling from our study revealed that glutamine and glutamate were abundant amino acids in chondrosarcomas and were elevated in mutant *IDH* chondrosarcomas, suggesting that there is increased dependence on glutaminolysis. This metabolic alteration has been identified in mutant *IDH* solid tumors [4, 37–39] and has led to two clinical trials targeting inhibition of glutaminolysis. Thus, our amino acid profile of mutant *IDH* chondrosarcomas confirms elevated levels of glutamine and glutamate and the notion that a greater dependence on glutaminolysis is required to support energy production in chondrosarcoma.

Similar to our findings in chondrosarcomas, mutant *IDH* gliomas also show a global elevation of free amino acids and lipid precursors [49]. While findings in gliomas show depletion of the late-stage TCA cycle intermediates, fumarate and malate [49], we did not observe this in chondrosarcoma. This finding could be a result of enhanced amino acid metabolism aiding in the replenishment of late-stage TCA cycle intermediates and maintenance of carbon cycling in mutant *IDH* chondrosarcomas. Fumarate is replenished by phenylalanine and tyrosine, both of which are amino acids elevated in *IDH* mutant chondrosarcomas, and malate is derived from pyruvate, an organic acid which also showed a trend for increase in mutant *IDH* chondrosarcomas. Moreover, opposing metabolic findings from Reitman et al. may be explained due to a different cell type being profiled and the *in vitro* conditions of their study [49].

We found distinct acylcarnitine derangements in mutant *IDH* versus non-mutant chondrosarcomas. Our analysis suggested that lipid and amino acid-derived acylcarnitine species were elevated in mutant *IDH* chondrosarcomas. This could suggest that in response to mutation of the *IDH* enzyme, chondrosarcomas resort to a greater dependence on fatty acid oxidation and amino acid metabolism to maintain tumor cell energetics. Studies in skeletal muscle reported that accumulation of mitochondrial acylcarnitine species can also be a signature of mitochondrial lipid overload or ineffective mitochondrial lipid catabolism [14]. Thus, it remains unclear if the abundant acylcarnitine levels in mutant *IDH* chondrosarcomas are a signature of enhanced influx of acylcarnitines in the mitochondria from increased mitochondrial fatty acid catabolism or from “incomplete” fatty acid oxidation. To investigate this phenomenon more comprehensively, a future point of interest would be to assess mutant *IDH* chondrosarcoma mitochondria β-oxidative capacity and acylcarnitine flux metabolism by carbon isotope labeling.

Future directions of study include addressing the elevation of specific amino acid species and their associated roles in neoplastic biochemical pathways. For instance, glycine was the most abundant amino acid in chondrosarcomas and was elevated in mutant *IDH* chondrosarcomas. Serine was also found in elevated levels in mutant *IDH* chondrosarcomas. Glycine and serine are biosynthetically linked and aid in the synthesis of proteins, nucleic acids, and lipids crucial to cancer cell growth [50, 51]. Investigation of the association of serine and glycine with tumorigenesis and malignancy [50, 51] is an attractive candidate for future studies in mutant *IDH* chondrosarcomas.

Our study also revealed elevated BCAAs (valine, leucine, and isoleucine) levels in mutant *IDH* chondrosarcomas. BCAAs were recently found to be a source of carbon for fatty acid biosynthesis in neoplastic proliferation, and suppression of BCAA metabolism led to a reduction in fatty acid levels [42, 52]. This interesting interplay between BCAA metabolism and fatty acid metabolism is another attractive candidate for further investigation in mutant *IDH* chondrosarcomas.

## Conclusions

Our metabolomics profiling of 17 human chondrosarcomas reveals a number of distinct alterations in mutant *IDH* chondrosarcomas including differences in glycolytic intermediates, TCA cycle intermediates, amino acids, and acylcarnitines levels. This study revealed that mutant *IDH* chondrosarcomas are metabolically more active tumors compared with *IDH* non-mutant chondrosarcomas. While there are conflicting data on *IDH* mutational status and associated clinical outcome in chondrosarcomas [53, 54], our metabolomic profiling shows that mutant *IDH* chondrosarcomas are metabolically distinct and more active than non-mutant chondrosarcomas, giving rise to the potential that *IDH* mutational state alters cell behavior. The global elevation of amino acids, lactate accumulation, and elevation of acylcarnitines and eventual metabolic dependence of these metabolites for neoplastic energetics is a signature of *IDH* mutant chondrosarcomas. In support of our metabolomics findings, our analysis of previously published mRNA microarray data [24, 25] revealed increased gene expression levels of specific metabolism genes in mutant *IDH* chondrosarcomas, which was also found to correlate to poor patient survival.

## Supplementary Information

**Additional file 1: Supplemental Figure 1**. UMAP clustering analysis of metabolites from mutant *IDH* and non-mutant chondrosarcomas display strong spatial clustering in organic acid and amino acid groups but weak spatial clustering in acylcarnitine species. UMAP analysis was performed in each metabolite group A) 7 organic acids (5 TCA cycle and 2 glycolytic intermediates, B) 17 amino acids, C) 45 acylcarnitines, D) total 69 metabolites

**Additional file 2: Supplemental Figure 2**. ^13^C α-KG labelling is reduced in mutant *IDH1* and *IDH2* chondrosarcoma cells based on measurements of citrate and pyruvate labelling. ^13^C isotope labelling of α-KG was achieved by 6 hours [^13^C_6_] labelling of glucose. Data normalized from citrate (M2) and pyruvate (M3) labelling display effects of mutated *IDH1* and *IDH2* enzymes A) reduction of α-KG carbon labelling from M2 citrate in mutant *IDH* chondrosarcoma cells B) reduction of α-KG carbon labelling from M3 pyruvate in mutant *IDH* chondrosarcoma cells

**Additional file 3: Supplemental Figure 3**. Expression of genes regulating metabolism correlates with prognosis in chondrosarcomas. A number of metabolism genes were found to be A) elevated in mutant *IDH1* and/or mutant *IDH2* chondrosarcomas and B-H) were predicative of patient survival

## Data Availability

All data generated or analyzed during this study are included in this published article (and its supplementary information files).

## Abbreviations

IDH1: Isocitrate dehydrogenase 1
IDH2: Isocitrate dehydrogenase 2
UMAP: Uniform Manifold Approximation and Projection
TCA: Tricarboxylic acid
α-KG: α-Ketoglutarate
D-2HG: D-2-Hydroxyglutarate
HIF1α: Hypoxia-inducible factor 1a
GC/MS: Gas chromatography-mass spectrometry
MS/MS: Mass spectrometry
IRB: Institutional review board
BCAAs: Branched-chain amino acids
LDHA: Lactate dehydrogenase
