# Supplementary material for: Mutant IDH and non-mutant chondrosarcomas display distinct cellular metabolomes
Source: Cancer Metab. 2021 Mar 24;9:13. doi: 10.1186/s40170-021-00247-8 (PMC7992867; doi:10.1186/s40170-021-00247-8)
Supplement: Supplementary file 1 — Additional file 1: Supplemental Figure 1. UMAP clustering analysis of metabolites from mutant IDH and non-mutant chondrosarcomas display strong spatial clustering in organic acid and amino acid groups but weak spatial clustering in acylcarnitine species. UMAP analysis was performed in each metabolite group A) 7 organic acids (5 TCA cycle and 2 glycolytic intermediates, B) 17 amino acids, C) 45 acylcarnitines, D) total 69 metabolites [file 40170_2021_247_MOESM1_ESM.docx]

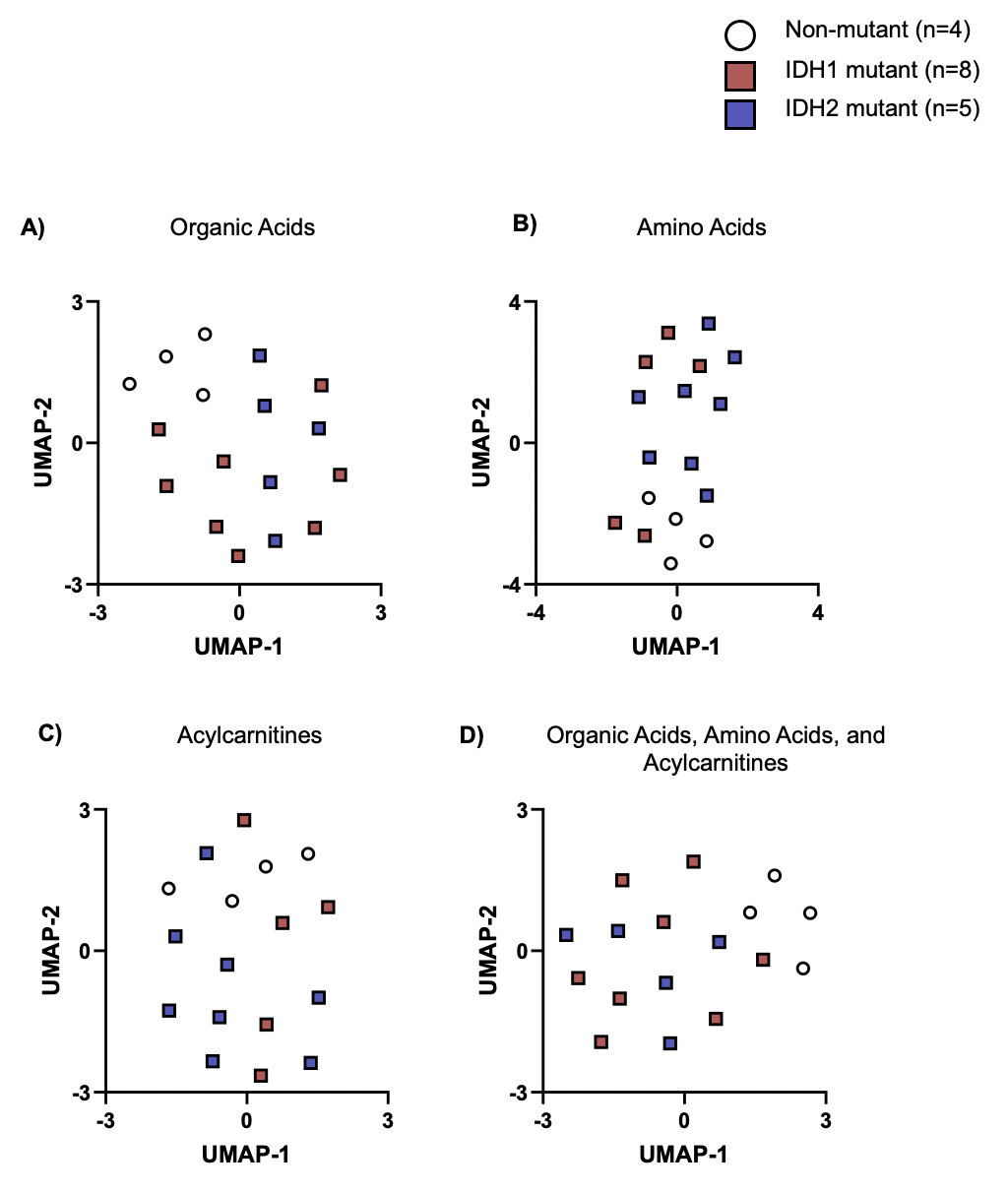


**Supplemental Figure 1. UMAP clustering analysis of metabolites from mutant *IDH* and non-mutant chondrosarcomas display strong spatial clustering in organic acid and amino acid groups but weak spatial clustering in acylcarnitine species.** UMAP analysis was performed in each metabolite group A) 7 organic acids (5 TCA cycle and 2 glycolytic intermediates, B) 17 amino acids, C) 45 acylcarnitines, D) total 69 metabolites.
