# Supplementary material for: Mutant IDH and non-mutant chondrosarcomas display distinct cellular metabolomes
Source: Cancer Metab. 2021 Mar 24;9:13. doi: 10.1186/s40170-021-00247-8 (PMC7992867; doi:10.1186/s40170-021-00247-8)
Supplement: Supplementary file 2 — Additional file 2: Supplemental Figure 2. 13C α-KG labelling is reduced in mutant IDH1 and IDH2 chondrosarcoma cells based on measurements of citrate and pyruvate labelling. 13C isotope labelling of α-KG was achieved by 6 hours [13C6] labelling of glucose. Data normalized from citrate (M2) and pyruvate (M3) labelling display effects of mutated IDH1 and IDH2 enzymes A) reduction of α-KG carbon labelling from M2 citrate in mutant IDH chondrosarcoma cells B) reduction of α-KG carbon labelling from M3 pyruvate in mutant IDH chondrosarcoma cells [file 40170_2021_247_MOESM2_ESM.docx]

**A)** **B)**

**Supplemental Figure 2.** **^13^C α-KG labelling is reduced in mutant *IDH1* and *IDH2* chondrosarcoma cells based on measurements of citrate and pyruvate labelling.** ^13^C isotope labelling of α-KG was achieved by 6 hours [^13^C_6_] labelling of glucose. Data normalized from citrate (M2) and pyruvate (M3) labelling display effects of mutated *IDH1* and *IDH2* enzymes **A)** reduction of α-KG carbon labelling from M2 citrate in mutant *IDH* chondrosarcoma cells **B)** reduction of α-KG carbon labelling from M3 pyruvate in mutant *IDH* chondrosarcoma cells.
