# Supplementary material for: Mutant IDH and non-mutant chondrosarcomas display distinct cellular metabolomes
Source: Cancer Metab. 2021 Mar 24;9:13. doi: 10.1186/s40170-021-00247-8 (PMC7992867; doi:10.1186/s40170-021-00247-8)
Supplement: Supplementary file 3 — Additional file 3: Supplemental Figure 3. Expression of genes regulating metabolism correlates with prognosis in chondrosarcomas. A number of metabolism genes were found to be A) elevated in mutant IDH1 and/or mutant IDH2 chondrosarcomas and B-H) were predicative of patient survival [file 40170_2021_247_MOESM3_ESM.docx]

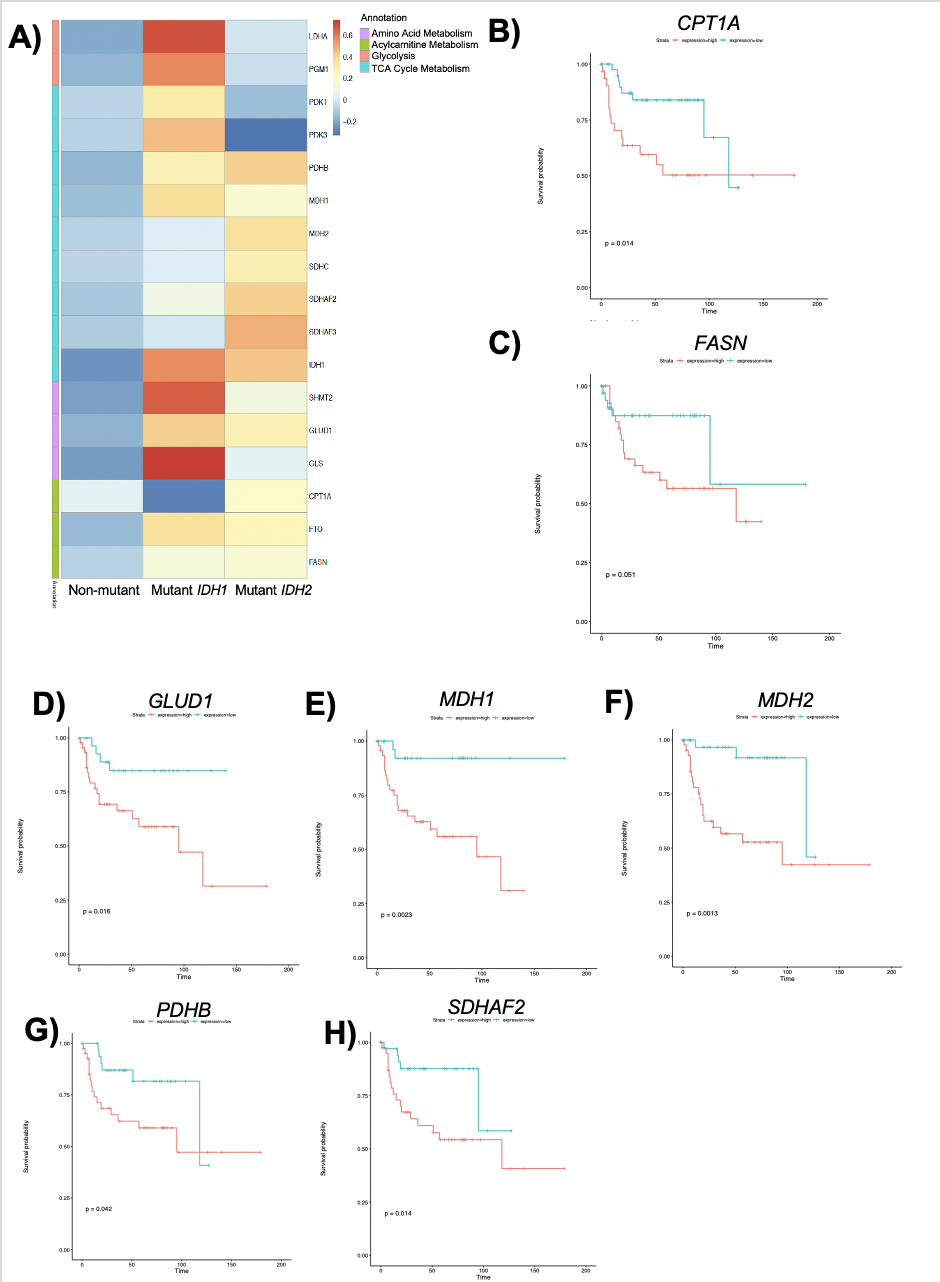
**Supplemental Figure 3. Expression of genes regulating metabolism correlates with prognosis in chondrosarcomas.** A number of metabolism genes were found to be A) elevated in mutant *IDH1* and/or mutant *IDH2* chondrosarcomas and B-H) were predicative of patient survival.
